# Additive Manufactured Poly(ε-caprolactone)-graphene Scaffolds: Lamellar Crystal Orientation, Mechanical Properties and Biological Performance

**DOI:** 10.3390/polym14091669

**Published:** 2022-04-20

**Authors:** Sara Biscaia, João C. Silva, Carla Moura, Tânia Viana, Ana Tojeira, Geoffrey R. Mitchell, Paula Pascoal-Faria, Frederico Castelo Ferreira, Nuno Alves

**Affiliations:** 1 Centre for Rapid and Sustainable Product Development (CDRSP), Polytechnic of Leiria, Marinha Grande, 2430-028 Leiria, Portugal; sara.biscaia@ipleiria.pt (S.B.); joao.f.da.silva@tecnico.ulisboa.pt (J.C.S.); carla.moura@ipleiria.pt (C.M.); taniaviana@gmail.com (T.V.); anatojeira88@gmail.com (A.T.); geoffrey.mitchell@ipleiria.pt (G.R.M.); paula.faria@ipleiria.pt (P.P.-F.); 2 Department of Bioengineering and IBB—Institute for Bioengineering and Biosciences, Instituto Superior Técnico, Universidade de Lisboa, Av. Rovisco Pais, 1049-001 Lisboa, Portugal; frederico.ferreira@tecnico.ulisboa.pt; 3 Associate Laboratory I4HB—Institute for Health and Bioeconomy, Instituto Superior Técnico, Universidade de Lisboa, Av. Rovisco Pais, 1049-001 Lisboa, Portugal

**Keywords:** additive manufacturing, graphene, lamellar crystal orientation, mechanical properties, poly(ε-caprolactone), tissue engineering

## Abstract

Understanding the mechano–biological coupling mechanisms of biomaterials for tissue engineering is of major importance to assure proper scaffold performance in situ. Therefore, it is of paramount importance to establish correlations between biomaterials, their processing conditions, and their mechanical behaviour, as well as their biological performance. With this work, it was possible to infer a correlation between the addition of graphene nanoparticles (GPN) in a concentration of 0.25, 0.5, and 0.75% (*w*/*w*) (GPN0.25, GPN0.5, and GPN0.75, respectively) in three-dimensional poly(ε-caprolactone) (PCL)-based scaffolds, the extrusion-based processing parameters, and the lamellar crystal orientation through small-angle X-ray scattering experiments of extruded samples of PCL and PCL/GPN. Results revealed a significant impact on the scaffold’s mechanical properties to a maximum of 0.5% of GPN content, with a significant improvement in the compressive modulus of 59 MPa to 93 MPa. In vitro cell culture experiments showed the scaffold’s ability to support the adhesion and proliferation of L929 fibroblasts (fold increase of 28, 22, 23, and 13 at day 13 (in relation to day 1) for PCL, GPN0.25, GPN0.5, and GPN0.75, respectively) and bone marrow mesenchymal stem/stromal cells (seven-fold increase for all sample groups at day 21 in relation to day 1). Moreover, the cells maintained high viability, regular morphology, and migration capacity in all the different experimental groups, assuring the potential of PCL/GPN scaffolds for tissue engineering (TE) applications.

## 1. Introduction

TE is a multidisciplinary scientific field that combines cells, biomaterial scaffolds, and physical/chemical cues with the aim of developing biological substitutes to overcome the significant shortage of tissues and organs for transplantation. Due to the scaffold’s importance as structural support for cell growth and differentiation, a broad variety of biomaterials and conventional scaffold fabrication methods have been investigated [1,2,3].

TE strategies must provide a high compatibility with cell adhesion and proliferation, which is a major requirement for proper tissue and organ formation. Such engineered tissues have high potential to improve quality of life, reducing patient morbidity and mortality. However, conventional fabrication technologies are limited in their ability to create clinically applicable tissue constructs with control over pore size and distribution, reproducibility, and scalability [4,5].

Additive manufacturing (AM), sometimes referred to as three-dimensional (3D) printing, represents a new group of non-conventional techniques recently introduced into the medical field. These techniques enable a high degree of reproducibility and the capacity to quickly produce complex 3D structures with an elevated level of control over a predefined geometry, size, and interconnectivity between pores, which promotes vascularisation and diffusion of oxygen and nutrients throughout the scaffold and provides a biomechanical environment adequate for tissue regeneration [6,7,8].

Polymer nanocomposites with nano-sized carbonaceous fillers have attracted attention due to improvements in mechanical properties and conductivity when compared to the original polymer [9,10]. The development of well-dispersed graphene nanoparticles (GPN) in a polymeric matrix has opened a new and interesting topic in materials science [11]. 

Graphene is a monolayer of carbon atoms arranged in a two-dimensional (2D) honeycomb lattice structure that has gained special interest by the scientific community due to its advantageous mechanical, electrical, and thermal properties [12]. We follow the draft proposal of the Graphene Council [13] that graphene refers to a material “composed of one or more layers/sheets of sp²-bonded carbon atoms in a crystal lattice”. As result of their unique properties, GPN demonstrate great potential for many applications, such as electronics, energy or hydrogen storage, and sensing [14]. Recent studies have shown that GPN also have significant potential for biomedical applications, such as drug delivery, cancer therapy, biological sensing, antibacterial material, and biocompatible scaffold manufacture [15]. The addition of small amounts of this electrically conducting filler reinforces the mechanical, thermal, and electrical properties of biocompatible polymers, for example PCL, compared to the neat polymer, without compromising their biocompatibility [15,16,17,18]. 

PCL is a linear aliphatic polyester that has been reported to be a promising polymer for biomedical applications because it is Food and Drug Administration (FDA) approved, assuring its biocompatibility and biodegradability properties. In addition, PCL exhibits good mechanical properties with high flexibility, great elongation at break, and low melting point (~60 °C), which is advantageous for 3D printing of scaffolds [19,20].

The combination of PCL with GPN has been studied for many different biomedical applications, including their interaction with different cell types [15,21,22,23]. Recently, some research groups started using this type of nano-composite to fabricate 3D printed scaffolds for bone TE [24,25]. However, no previous work has studied the orientation of the lamellar crystals of 3D PCL/GPN printed scaffolds through small-angle X-ray scattering (SAXS) experiments. SAXS is a powerful technique for evaluating the molecular organisation of materials, including polymers. These experiments have been conducted to extract information regarding the long period, which, with knowledge of the degree of crystallinity, can be used to calculate the crystalline and amorphous thickness from SAXS data [26]. The use of SAXS as a characterization technique for polymeric materials has been extended to included studies of polymer blends and nanocomposite materials [26,27]. Additionally, in this work we analysed the impact of processing conditions on internal morphology of the filaments and their effects on the scaffold’s thermal, mechanical, and biological properties. Thus, the focus of this work is the investigation of these properties while evaluating the impact of the addition of different quantities of GPN in 3D PCL scaffolds, which is of major importance to assure their performance in situ. 

## 2. Materials and Methods

### 2.1. Materials

PCL spheres (Ø~3 mm; MW 50,000) were sourced from Perstorp (Cheshire, UK). Graphene nano-flakes (average flake thickness 12 nm; purity 99.2%; average particle (lateral) size 4500 nm (1500–10,000) nm; grade AO-3) were obtained from Graphene Supermarket (Graphene Laboratories Inc., Calverton, NY, USA). *N*,*N*-dimethylformamide (DMF) from Merck KGaA (Darmstadt, Germany) was used as solvent to obtain PCL/GPN composite. Chloroform from Sigma-Aldrich was used as solvent to produce films for electrical conductivity measurements. Regarding the in vitro tests, the following chemicals were used: Dulbecco’s Modified Eagle’s Medium (DMEM, Thermo Fisher Scientific, Waltham, MA, USA); Fetal Bovine Serum (FBS, Gibco, Thermo Fisher Scientific, Waltham, MA, USA); antibiotic–antimycotic (Anti–Anti, Gibco, Thermo Fisher Scientific, Waltham, MA, USA); MTT (In vitro Toxicology Assay Kit MTT based from Sigma-Aldrich); anhydrous isopropanol (Sigma-Aldrich, St. Louis, MO, USA); phosphate buffered saline (PBS, Gibco, Thermo Fisher Scientific, Waltham, MA, USA); AlamarBlue^®^ assay (Thermo Fisher Scientific, Waltham, MA, USA); LIVE/DEAD kit (Thermo Fisher Scientific, Waltham, MA, USA); paraformaldehyde (PFA, Sigma-Aldrich, St. Louis, MO, USA); phalloidin-TRITC (Sigma-Aldrich, St. Louis, MO, USA; 2 μg/mL, stains actin-rich cytoskeleton red); Triton X-100 (Sigma-Aldrich, St. Louis, MO, USA); DAPI (Sigma-Aldrich, St. Louis, MO, USA; 1.5 μg/mL, stains cell nuclei blue).

### 2.2. Preparation of PCL/GPN Composite

PCL/GPN composites with 0.25, 0.50, and 0.75 wt% GPN composition (represented as GPN0.25, GPN0.50, and GPN0.75, respectively) were prepared by dissolution of previously weighted PCL (30 g) in 120 mL of DMF and stirred at 50 °C for 30 min. After full dissolution of the PCL, GPN were dispersed in the solution with a constant agitation for 1 h to assure good dispersion and homogeneity. Following, the solution was deposited into petri dishes and left to dry in a laboratory fume hood until complete solvent evaporation. The obtained samples with ≈2 mm thickness were then sliced into small squares, for further use. 

### 2.3. Scaffold Design and Fabrication

**Figure 1:**
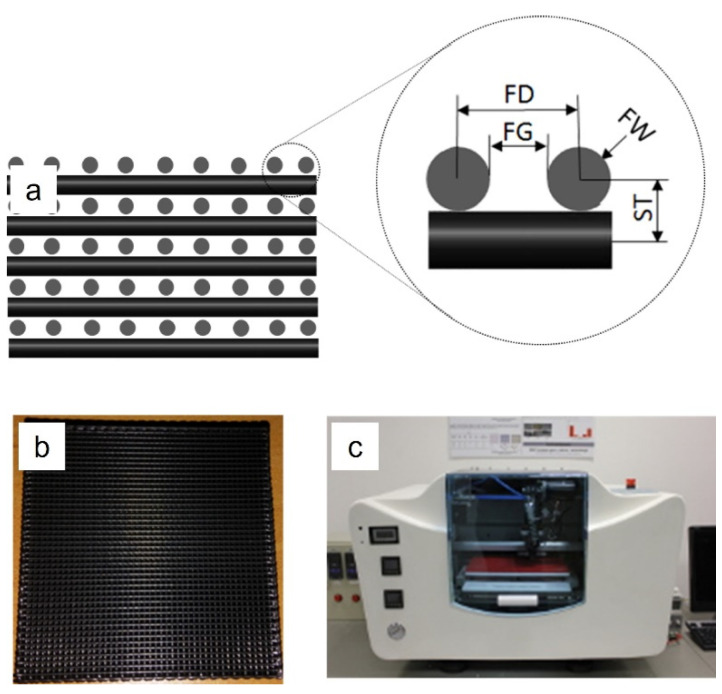
Scaffold design and fabrication; (**a**) cross-section of a 3D PCL/GPN design and its design parameters (FG, FD, FW, and ST); (**b**) 3D PCL/GPN scaffold, fabricated using the (**c**) Bioextruder^®^ system (CDRSP homemade extrusion-based AM system).

3D PCL/GPN scaffolds were fabricated using the fused deposition modelling (FDM) technique with the Bioextruder, a biomanufacturing system developed at Centre for Rapid and Sustainable Product Development of the Polytechnic of Leiria (Leiria, Portugal) [28,29,30,31]. This is a pellet-fed-extruder-based system. The 3D scaffolds (30 (length) × 30 (width) × 8 (height) mm) were designed adopting a 0/90° lay-down pattern and the following design parameters: filament gap (FG), filament distance (FD), filament width (FW), and slice thickness (ST) (Figure 1, Table 1).

### 2.4. Characterisation of PCL/GPN Scaffolds

#### 2.4.1. Differential Scanning Calorimetry (DSC) and Thermogravimetric Analysis (TGA)

The thermal properties of the PCL/GPN composites before and after processing by the bioextrusion technique were assessed using an STA 6000 (Perkin Elmer^®^, Waltham, MA, USA). All operations were performed under a nitrogen atmosphere with a flowrate of 20 mL/min and the weight of the samples varied between 6–7 mg. Each sample was heated first to 120 °C at 10 °C/min and then held at that temperature for 1 min to erase any thermal history. The samples were cooled to 15 °C (5 min hold) and heated to 120 °C (all steps were at 10 °C/min). The degree of crystallinity, *X_c_*, of PCL and the PCL/GPN based composites were calculated from the areas of the corresponding DSC melting peaks using Equation (1) [32]:

(1) $$X_{c}=\frac{\Delta H_{m}}{\Delta H_{0}\times X_{PCL}}\times 100$$

where *X_PCL_* is the weight fraction of PCL in the composite, Δ*H*_0_ is the heat of fusion of 100% crystalline PCL (139.5 J/g [33,34]), and Δ*H_m_* is the peak area of the melting range considered. To evaluate the thermal degradation of the materials, the samples were exposed to a temperature ramp from 30 °C to 600 °C at a heating rate of 10 °C/min.

#### 2.4.2. Measurement of the Electrical Conductivity of the PCL/GPN Composites

GPN0.25, GPN0.50, and GPN0.75 films were produced by dissolving the different composites in chloroform at 10 wt% in glass plates and by promoting solvent evaporation overnight inside a laboratory fume hood. For the electrical conductivity measurements, the films were coated with four 50 nm thick gold strips using an e-beam evaporator (Temescal, Ferrotec, Santa Clara, CA, USA) to improve electrical contact. The D.C. electrical conductivity of the composites was measured using the 4-point probe technique (Suss Manual 4 Probe station coupled to Keithley 4200 IV/CV meter, Tektronix, Beaverton, OR, USA). From previous tests, this equipment is able to measure electrical conductivities above 10^−7^ S/m [35,36]. 

#### 2.4.3. Surface Hydrophilicity Characterization

The wettability of the PCL/GPN scaffolds was determined through measurement of the static contact angle at room temperature by using a Theta Lite optical tensiometer (Attension, Biolin Scientific, Stockholm, Sweden). A ~10 µL deionized water droplet was deposited onto the scaffold (*n* = 7), the droplet was imaged by a high-speed camera after 10 s, and the contact angle was measured by OneAttension 1.0. software (Biolin Scientific, Stockholm, Sweden).

#### 2.4.4. Mechanical Testing of the 3D PCL/GPN Scaffolds

Compression tests were conducted to evaluate the mechanical behaviour of the fabricated scaffolds. The mechanical properties were determined using a ZWICK Z100 (ZwickRoell, Ulm, Germany) testing system, with a cross-head speed of 1 mm/min until a strain value of 0.5. Samples were characterized in the dry state with dimensions of 4 mm length, 4 mm width and 8 mm height. The stress was evaluated as the load divided by the area of the cross section of the scaffold. The strain was defined as the ratio between the scaffold height variation and the scaffold initial height. The compressive modulus was calculated from the slope of the initial linear region of the stress–strain curve.

#### 2.4.5. SAXS Experiments

SAXS experiments were carried out using the beamline NCD at the ALBA Synchrotron Light Facility (Barcelona, Spain). This beam line is a variable wavelength beam line using a vacuum undulator as the source. It was equipped with a ADSC Q210r CCD detector (pixel size 51 µm × 51 µm) for 2D SAXS patterns acquisition with a sample-to-detector distance of ~6 m and an incident x-ray wavelength of 1.0 Å and a LX255-HS CCD detector from Rayonix LLC for 2d wide-angle x-ray scattering, although these data are not reported here. Calibration was performed using silver behenate and chromium dioxide samples. The data accumulation time was 5 s, and the incident beam size had a width of 300 µm and a height of 200 µm.

### 2.5. PCL/GPN Scaffold Biological Performance

#### 2.5.1. In Vitro Cytotoxicity Evaluation

The biocompatibility of the different PCL/GPN composites was assessed using L929 mouse fibroblasts to evaluate possible cytotoxic effects of the different 3D scaffolds according to the guidelines of the ISO 10993-5:2009 (Biological evaluation of medical devices—Part 5: Tests for in vitro cytotoxicity) [37]. Prior to the test, all scaffolds were placed in a 70% ethanol solution and exposed to UV light overnight to assure their sterilization. All materials were assessed by performing indirect extract and direct contact in vitro cytotoxicity tests. L929 fibroblasts cultured on tissue culture polystyrene (TCPS) plates with DMEM supplemented with 10% (*v*/*v*) FBS and 1% Anti–Anti in an incubator at 37 °C/5% CO_2_ were used as negative control, and latex was used as positive control for cytotoxic effects. Fibroblasts, 1 × 10^5^ cells/well, were seeded on TCPS plates and cultured for 24 h at 37 °C and 5% CO_2_ to obtain a confluent monolayer. For the indirect extract test, the culture media was removed and L929 cells were exposed to the material extract’s conditioned medium for 72 h at 37 °C/5% CO_2_. Then, extract-conditioned mediums were removed, and the MTT (3-(4,5-dimethylthiazol-2-yl)-2-5 diphenyl tetrazolium bromide) assay was performed. Cells were incubated with MTT solution (1 mg/mL, yellow) for 2 h at 37 °C; afterwards, the violet formazan product resulting from the MTT metabolic reduction by metabolically active cells was dissolved under agitation using a 0.1 N HCl solution in anhydrous isopropanol. Absorbance values of the resultant solutions were measured in a plate reader (Infinite M200 PRO, TECAN, Männedorf, Switzerland) at 570 nm. For each condition, three samples were assessed, and the absorbance values of each sample were measured in triplicate. The percentage of viable cells for the different experimental groups was normalized to the negative control. For the direct contact test, scaffolds were placed on top of a confluent monolayer of L929 fibroblasts and incubated for 72 h at 37 °C/5% CO_2_. Afterwards, the morphology of L929 fibroblasts was evaluated qualitatively under an inverted optical microscope (LEICA DMI3000B, Leica Microsystems, Wetzlar, Germany) equipped with a digital camera (Nikon DXM1200F, Nikon Instruments Inc., Tokyo, Japan).

#### 2.5.2. Cell Seeding and Culture on 3D PCL/GPN Scaffolds

The ability of 3D porous PCL/GPN scaffolds to promote cell proliferation was studied using two different cell types: L929 mouse fibroblasts and human bone marrow-derived mesenchymal stem/stromal cells (BM MSCs). BM MSCs are the gold-standard cell source for cell therapy and tissue engineering applications [38,39], and, as the 3D PCL/GPN scaffolds presented in this study were fabricated envisaging possible applications in the regeneration of electroactive hard tissues such as bone, BM MSCs are particularly advantageous because of their role as bone progenitor cells present on native bone tissue within the bone marrow and due to their high osteogenic potential, which make them able to efficiently differentiate into bone lineage cells (osteoblasts and osteocytes) [40,41].

Prior to cell culture studies, the scaffolds were sterilized in ethanol 70% washing and by UV exposure overnight. Afterwards, the scaffolds were rinsed three times with a 1% Anti–Anti solution prepared in PBS for 3 h and incubated in culture medium for 1 h. For the studies with L929 fibroblasts, the cells were seeded onto the scaffolds (8 × 10^4^ cells/scaffold) that had been previously placed in a 24-well plate ultra-low attachment (Corning). Scaffolds were then incubated for 1.5 h without culture medium to allow initial cell attachment. Culture medium was added to each scaffold, and the cultures were maintained for 13 days at 37 °C/5% CO_2_ with full renewal of the culture medium every 2–3 days.

BM MSCs were isolated from bone marrow aspirates (male donor of 35 years age), after informed consent and with the approval of the ethics committee of Instituto Português de Oncologia Francisco Gentil (Lisbon, Portugal), and characterized following protocols previously developed by our group [42,43]. BM MSCs were seeded onto the scaffolds placed in a 24-well plate ultra-low attachment at a density of 1 × 10^5^ cells per scaffold and incubated for 1.5 h without medium to promote initial cell adhesion. Then, DMEM supplemented with 10% FBS and 1% Anti–Anti medium was added to the scaffolds. The BM MSCs-seeded scaffolds of the different experimental groups were cultured for 21 days at 37 °C/5% CO_2_ in an incubator, and medium renewal was performed each 2–3 days.

#### 2.5.3. Cell Proliferation (Alamar Blue) Assay

The metabolic activity of cells in the different experimental scaffold groups was evaluated on days 1, 5, 9, and 13 for L929 fibroblasts; and on days 1, 7, 14, and 21 for BM MSCs using the AlamarBlue^®^ assay according to the manufacturer’s guidelines. Briefly, scaffold samples were incubated with a 10% *v*/*v* AlamarBlue solution (prepared in the respective culture medium) for 2.5 h at 37 °C/5% CO_2_. Afterwards, the solution fluorescence intensity was measured in a plate fluorometer (Infinite^®^200 PRO, TECAN, Männedorf, Switzerland) with an excitation wavelength of 560 nm and emission wavelength of 590 nm. Three scaffolds (*n* = 3) were considered for each experimental group, and the florescence intensity values for each scaffold sample were measured in triplicate. Scaffolds without cells were used as blank controls. Equivalent cell numbers were estimated using as calibration (one for each specific cell type) the correlation between measured fluorescence intensity values of AlamarBlue^®^ assay with counted cells (trypan blue exclusion method) cultured in standard 24-well TCPS plates of tissue culture polystyrene (BD Falcon^®^, Corning, New York, NY, USA).

#### 2.5.4. BM MSC Viability and Morphological Analysis

The viability of BM MSCs in the final constructs (at day 21) was analysed using a LIVE/DEAD kit. Samples were incubated in PBS containing 2 μM calcein AM (stains live cells green) and 4 μM ethidium homodimer-1 (stains dead cells red) for 1 h at room temperature, washed with PBS, and immediately imaged under a fluorescence microscope (LEICA DMI3000B, Leica Microsystems, Wetzlar, Germany).

The morphology of BM MSCs after 21 days of culture in the different scaffold experimental groups was assessed through DAPI/phalloidin (DAPI/PHA) fluorescent staining. For that, the samples were washed twice with PBS, fixed with 4% *v*/*v* PFA solution, and then permeabilized with 0.1% Triton X-100 solution for 10 min. Afterwards, samples were incubated with Phalloidin-TRITC) for 45 min in the dark, washed twice with PBS, and counterstained with DAPI. The samples were then washed with PBS and imaged by fluorescence microscopy (LEICA DMI3000B, Leica Microsystems, Germany).

BM MSCs morphology on the porous scaffolds at day 21 was also observed using scanning electron microscopy (SEM) (Hitachi model S2400, Hitachi, Japan). Fixed samples were dehydrated using ethanol gradient solutions (20%, 40%, 60%, 80%, 95%, and 100% (*v*/*v*); 20 min incubation with each solution). Finally, the dried cell–scaffold samples were sputter-coated (Quorum Technologies model E5100, Sussex, UK) with a thin layer of gold–palladium and imaged.

### 2.6. Statistical Analysis

All experimental data were statistically analysed by GraphPad Prism 9 and are presented as mean ± SD. The statistical analysis of mechanical tests was performed using one-way analysis of variance (ANOVA) with Tukey multi-comparison test. Differences were considered statistically significant at *p* ≤ 0.05. Results of significance were considered as: * *p* < 0.05, ** *p* < 0.01, *** *p* < 0.001 and **** *p* < 0.0001. Two-way ANOVA was carried out in in vitro tests, and significant differences were recorded at a confidence level of 95%. Uncorrected Fisher’s LSD test was used for multi-comparisons. Replicates of each type of sample (at least *n* = 3) were performed.

## 3. Results

### 3.1. Processing Parameter Impact on Material Properties

**Figure 2:**
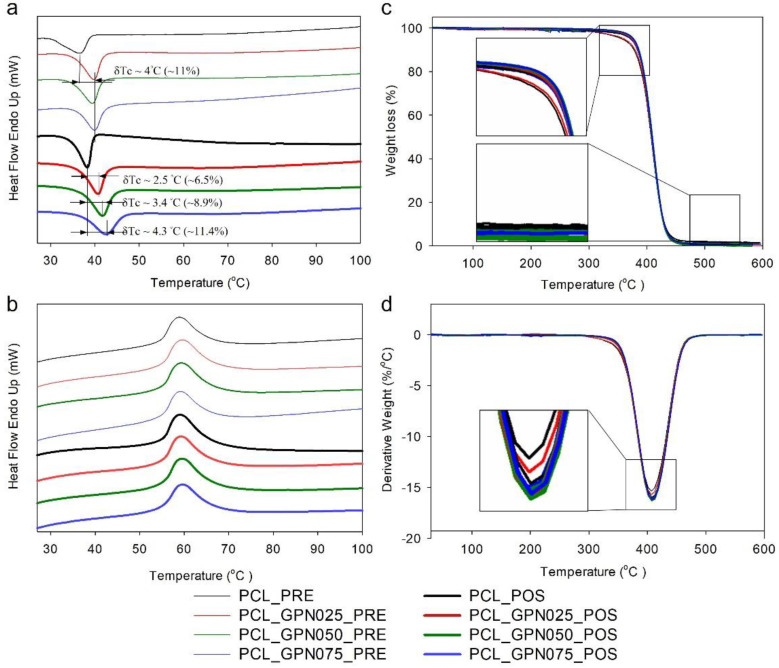
DSC (**left** column) and TGA (**right** column) results of the pre- and post-processed PCL/GPN composites: (**a**) DSC of first cooling cycle, (**b**) DSC of second heating cycle, (**c**) TGA curves, and (**d**) derivative of TGA curves.

DSC/TGA analyses were performed on the materials before (pre) and after (pos) processing. Figure 2 shows DSC and TGA thermograms of all the materials, and the main data is reported in Table 2. The crystallization temperature (*T_c_*) and melting point (*T_m_*) were determined as the peak temperatures of the first cooling scan curve (Figure 2a) and the second heating scan curve (Figure 2b), respectively. Upon material processing, *T_c_* and *P DTG* (Figure 2d) increased by about 3–7% and 0.2–1.1% (*p* < 0.05), respectively. Interestingly, regarding *T_c_*, the percentage of this increase was higher and quite similar in PCL and GPN0.75 samples, which is in accordance with other results discussed further. Regarding the influence of the addition of GPN, results detailed in Table 2 reveal a similar increase of *T_c_* for all GPN contents (~11%) relative to neat PCL before material manipulation. However, after processing, GPN content had an impact on *T_c_*, increasing it about 6.5%, 8.9%, and 11.4% for GPN0.25, GPN0.50, and GPN0.75, respectively. No statistical differences were noticed regarding *T_m_*. All composites showed similar melting peaks to neat PCL at ~59 °C.

The thermal stability of the samples was investigated by TGA. The pure thermoplastic and composites revealed a weight loss (Δ*M*) (Figure 2c) around 99% and a P DTG (Figure 2d) of ~411 °C. All composites showed very similar curves and a single weight loss step.

### 3.2. Impact of GPN on Scaffold Properties

**Figure 3:**
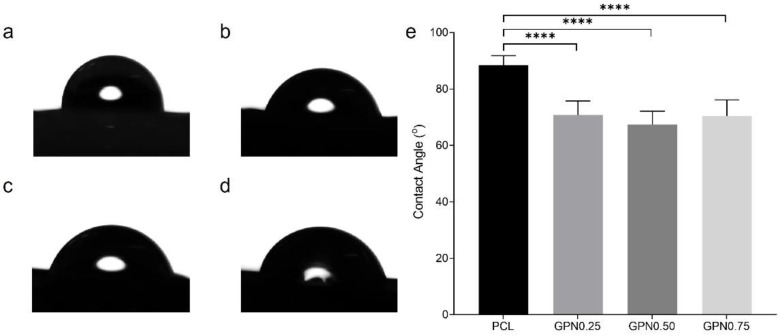
Contact angle images of the water droplet on the scaffold with (**a**) PCL, (**b**) GPN0.25, (**c**) GPN0.50, and (**d**) GPN0.75. (**e**) Contact angle measurements of the scaffolds. Statistical differences: **** *p* < 0.0001.

The water contact angle of PCL scaffolds was compared for all PCL/GPN groups (Figure 3). A water droplet with a contact angle of 88.41° was observed on the PCL scaffold’s surface. Regarding PCL/GPN composites, water contact angle was 70.80°, 67.42°, and 70.36° for GPN0.25, GPN0.50, and GPN0.75, respectively. The results revealed significant differences between PCL and PCL/GPN groups.

**Figure 4:**
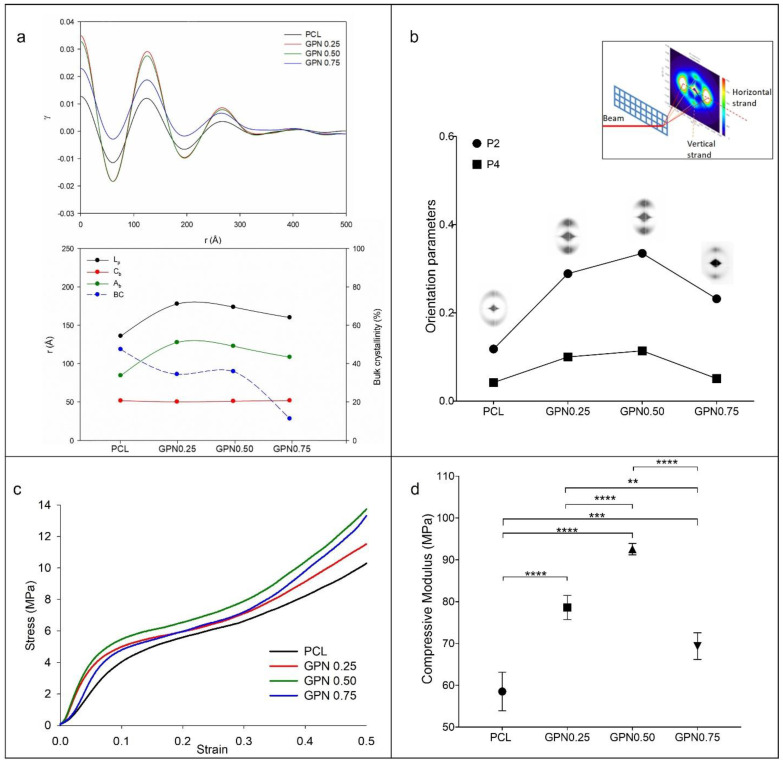
SAXS (upper line) and mechanical properties (bottom line): (**a**) Correlation function obtained from scattering curves (left), and variation in the values of long period (L_p_), crystalline (C_b_), and amorphous layer (A_b_) thicknesses and bulk crystallinity (BC) extracted from correlation functions with GPN content (right); (**b**) 2D SAXS pattern and orientation parameters <P2> and <P4> of PCL filaments with different contents on GPN; (**c**) PCL and PCL/GPN scaffold stress–strain curves under compression; (**d**) compressive modulus of 3D PCL and PCL/GPN scaffolds. Statistical differences: ** *p* < 0.01; *** *p* < 0.001; **** *p* < 0.0001.

The 2D SAXS patterns for each composite show typical patterns of a semi-crystalline polymer with a preferential orientation of the lamellar crystals (Figure 4a,b). There are two arcs above and below the zero-angle point, with maxima along the vertical axis. There is also some scattering around the zero-angle point and evidence of a thin horizontal streak that passes through the centre.

We have used the azimuthal variation in the intensity, *I*(*α*), at a constant value of the modulus of the scattering vector Q. The level of preferred orientation of the lamellae crystals was extracted using the expression shown below (Equation (2)) following the method developed by Mitchell and colleagues [44]. The orientation parameters <*P*_2_> and <*P*_4_> are the first two components of a series which describes the orientation distribution function of the normal to the lamellar crystals. If <*P*_2_> = 0 the distribution is isotropic, if <*P*_2_> = 1 the crystals are arranged with the same perfect preferred orientation.

(2) $${\langle P_{2n}\rangle }_{Q}=\frac{1}{P_{2}^{\;m}}\overset{\pi /2}{\underset{0}{\int }}\frac{I\left(\left|\underline{Q}\right|,\alpha \right)\sin\;\alpha P_{2}\;(\cos\alpha )\;d\alpha }{I\left(\left|\underline{Q}\right|,\alpha \right)\sin\alpha \;d\alpha }$$

Results are plotted in Figure 4b, which shows an increase in the level of preferred orientation with increasing level of GPN, but which falls off with the highest level of GPN (GPN0.75).

Correlation functions calculated from the scattering data using the methodology of Strobl and Schneider [45] and structural parameters of the lamellar crystals are presented in Figure 4a. Here, it is possible to observe that the lamellar crystals exhibit a constant thickness with the GPN variation content.

Figure 4c shows the representative mean stress and strain curves of PCL and PCL/GPN scaffolds. As expected, compressive modulus (Figure 4d) increases with the addition of GPN, as it acts as a structural reinforcement. However, and interestingly, there is a decrease of approximately 25% from GPN0.50 to GPN0.75.

### 3.3. Impact of GPN on Cell–Scaffold Interactions

Measurement of electrical conductivity of the strands within the composite films revealed that conductivity was less than 10^−7^ S/m.

**Figure 5:**
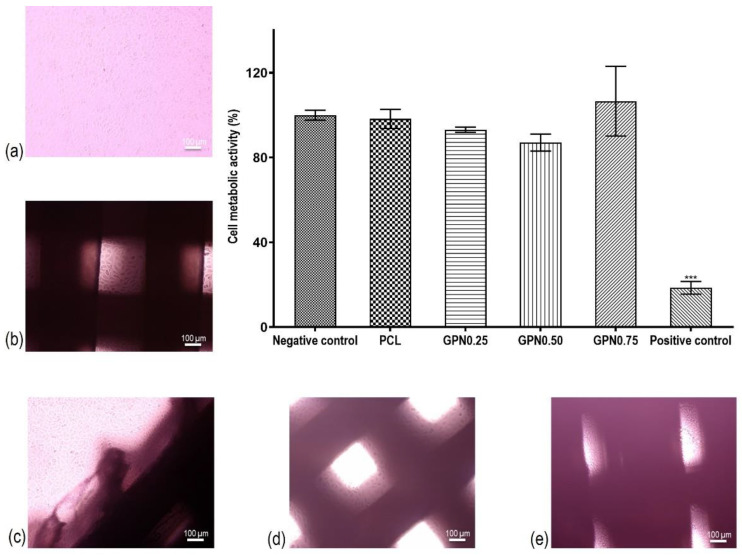
Biological in vitro testing of scaffolds: (**b**) PCL; (**c**) GPN0.25; (**d**) GPN0.50; (**e**) GPN0.75) with L929 cells; cytotoxicity assay according to ISO 10993-5 guidelines direct and indirect extract tests. The percentages of cell viability of the material extracts were normalized to the negative control (**a**) L929 fibroblasts in standard culture medium. The positive control used was latex for cytotoxic effects. Statistical differences: *** *p* < 0.001.

The results for the cytotoxicity assay according to ISO 10993-5 guidelines direct and indirect extract tests are shown in Figure 5. Results of the indirect extract assay showed cell viabilities higher than 85% for all conditions tested when compared to the control. A latex material was used to produce a cytotoxic response of cells to the material, showing high cell mortality, more than 80% upon exposure to toxic lixiviates driven from this material, confirming the sensitivity of the test (*p* < 0.001). The direct contact results corroborate the results obtained in the extract test, showing that L929 fibroblasts in contact with scaffolds maintain their regular morphology, and no evidence of cell death or halo inhibition were observed at the cell–scaffold interface.

**Figure 6:**
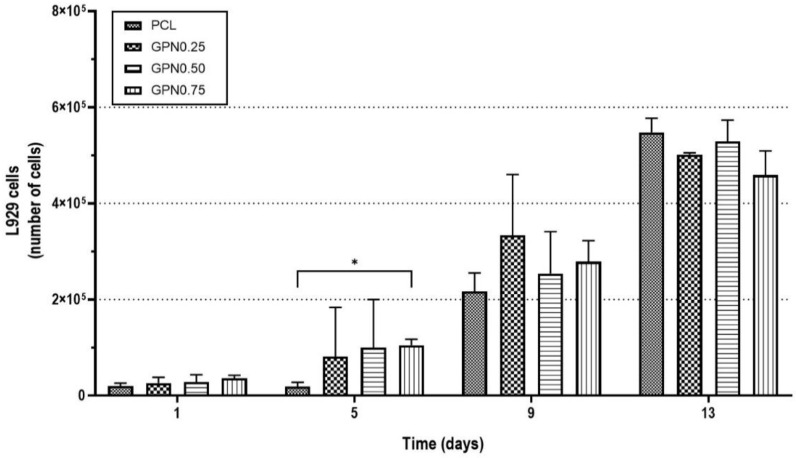
Biological in vitro testing of scaffolds with L929 fibroblasts: cell proliferation assessment. Two-way ANOVA, uncorrected Fisher’s LSD (*n* = 3), multiple comparisons. Statistical differences: * *p* < 0.05.

Moreover, a 13-day experiment was performed to further study cell–biomaterial interactions, namely, the scaffold’s capacity to promote L929 fibroblasts proliferation (Figure 6). It was possible to observe that L929 fibroblasts presented low levels of cell adhesion to the scaffolds, but with a high proliferation capacity with fold increase of 28, 22, 23, and 13 (day 13 in relation to day 1) of PCL, GPN0.25, GPN0.5, and GPN0.75, respectively.

**Figure 7:**
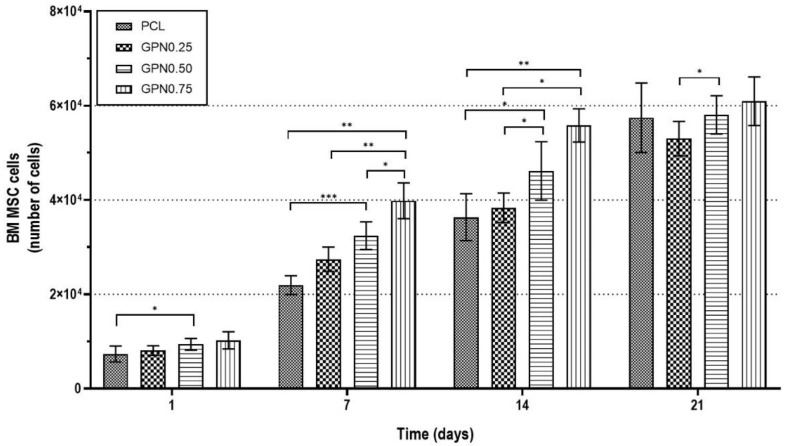
Biological in vitro testing of scaffolds with BM MSC: cell proliferation assessment. Two-way ANOVA, uncorrected Fisher’s LSD (*n* = 3), multiple comparisons. Statistical differences: * *p* < 0.05; ** *p* < 0.01; *** *p* < 0.001.

As observed with L929 fibroblasts, BM MSCs presented low cell adhesion to the material. However, they also presented a good proliferation capacity over the whole experiment, with approximately a seven-fold increase for all sample groups at day 21 in relation to day 1 (Figure 7). BM MSCs were present and covered almost all the structure, as it is possible to observe by DAPI/PHA staining and SEM analysis (Figure 6). Amplified images are presented in supplementary material (Figure S1).

**Figure 8:**
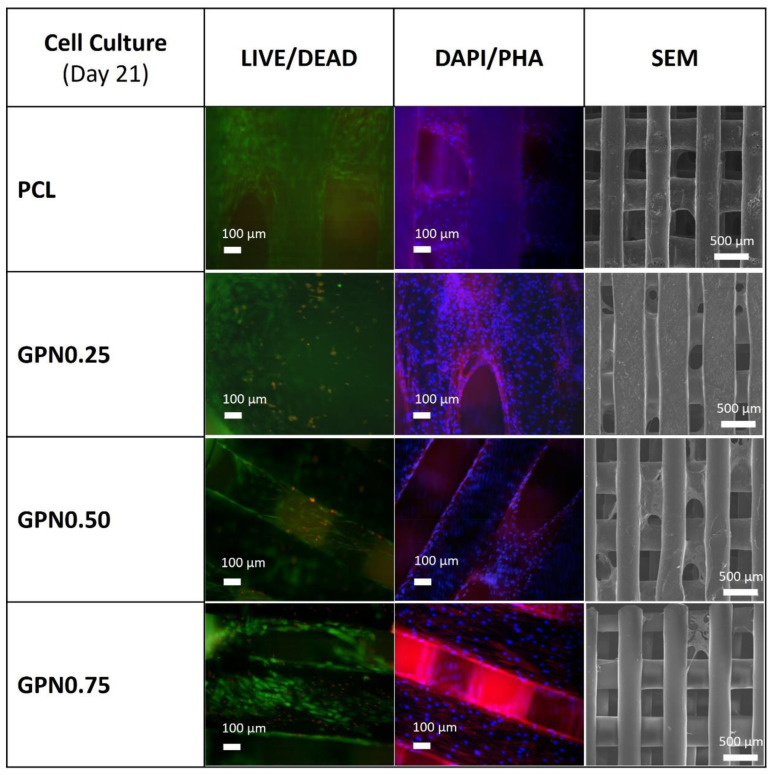
Biological in vitro testing of scaffolds with BM MSC: Cell viability, distribution and morphology assessment (at day 21) through LIVE/DEAD assay (first column), DAPI/PHA staining (second column), and SEM imaging (third column).

Although there are no significant statistical differences between PCL and PCL/GPN structures after three weeks (Figure 7), the results for 7 and 14 days show an increased number of cells with higher levels of GPN. We note that LIVE/DEAD staining revealed some level of cell death with the higher GPN content compared to the other conditions (Figure 8). Nevertheless, as can be observed for all scaffold groups in the DAPI/PHA staining and SEM analysis images (Figure 8), BM MSCs presented regular fibroblast-like spindle shape morphology and were able to migrate throughout the whole scaffold structure, even occupying its pores. These results together with the BM MSC proliferation capacity shown in Figure 7 suggest the suitability of 3D PCL/GPN scaffolds for tissue engineering applications.

## 4. Discussion

This work aims to understand the impact of the addition of GPN to a well-known and well-characterised FDA-approved polymer, PCL, but, at the same time and more importantly, to correlate the impact of processing conditions on material performance. To understand this impact, we should reflect on the possible consequences following the addition of GPN to a PCL matrix. The first is that if we add sufficient GPN, they can form a percolative pathway, the material will exhibit an electrical conductivity much above that of an insulator [34]. The addition of relatively small quantities of GPN may lead to a significant nucleating effect in the PCL matrix [12,35]. In the case of extruder-based 3D printing, *T_c_* is a critical parameter as it determines the morphology of the deposited material [36]. Moreover, if the graphene flakes adopt a preferential alignment in the extrudate, as has previously been observed [37], then this will result through the templating process in a preferred crystal alignment of the PCL lamellar crystals. Not least, the presence of GPN will have a reinforcing effect on the resultant composite.

Thermal analyses show that material-extrusion-based processing directly impacts *T_c_* and *P DTG*, which is in concordance with other works in the literature [46]. To understand the two effects shown in Figure 2a, we need to appreciate that the pre-processed samples and the post-processed samples have been crystallised in rather different conditions. The post-processed samples were extruded in the molten state at a specific temperature and under controlled conditions. Crystallisation takes place at a well-defined point as the extrudate cools after exiting the extruder [47]. In contrast, pre-processed material has crystallised in rather ill-defined conditions as the solvent evaporates, and clearly the nucleating effect of the graphene has a much-reduced impact. As reported in Table 2 regarding the effect of the addition of GPN, results reveal a similar increase of *T_c_* for all GPN contents (~11%) relative to neat PCL before material processing, which can be explained by the fact that in the solvent system higher concentrations of this filler may saturate its nucleating effect in the polymer. However, after processing, GPN content impacts *T_c_*, with a gradual increase in all nanocomposites (GPN0.25 6.5%, GPN0.50 8.9%, and GPN0.75 11.4%). This increment confirms that the addition of GPN enhances the nucleation of PCL crystallization [48,49]. All composites showed similar melting peaks, which at first glance suggests that scaffolds with or without the addition of GPN within the range studied can be produced with the same extrusion parameters. The thermal stability of all composites showed similar TGA curves, with one-step weight loss that corresponds to the decomposition of PCL [50,51]. The results reveal that the addition of GPN has no effect on the thermal behaviour of PCL [49,52]. Moreover, the processing temperature (80 °C) used to develop 3D scaffolds maintains the integrity of all the composites. Chung and colleagues [53] reported residual weight after the decomposition of the polymer attributed to graphene since there is minimal weight losses of this material in the evaluated temperature range [54]. However, in our results, after 450 °C, the remaining mass in all samples were very similar compared with pure PCL, as expected due to the small GPN content studied in this work. PCL and PCL/GPN scaffolds were successfully produced by extrusion-based AM process. The scaffolds presented the designed configurations, revealing the desired pore interconnectivity to assure scaffold mechanical integrity and, at the same time, assure the possibility of cell migration upon the whole scaffold. Surface wettability is an important factor in scaffold development since it affects the interaction between cells and biomaterials [55,56]. Therefore, assessment of the wettability of the scaffolds was performed by water contact angle in order to evaluate if material surfaces are hydrophilic (contact angle below 90°) or hydrophobic (contact angle above 90°). Contact angle results revealed that the addition of GPN caused scaffolds to become significantly more hydrophilic than pure PCL. However, no significant differences were observed between PCL/GPN groups. These results are in accordance with the literature, in which polymeric scaffolds increase hydrophilicity by GPN incorporation [52,57,58].

The 2D SAXS patterns in all four compositions show some scattering around the zero-angle point, and in the centre show a thin horizontal streak. We attribute this observation to voids present in the melt, which have been deformed into extended objects during extrusion. Such microvoids are common in fibres and extrudates [59]. The extent of arcing of these maxima is a measure of the preferred orientation in the lamellar crystals. The fact that the maxima are located on an axis parallel to the strand axis (and hence the extrusion direction) means that the lamellae are organised normal to that direction. We attribute this behaviour to flow through the extruder generating extended chains, which, if still present in the extrudate at the time of crystallisation, act as row nuclei, giving all the lamellar crystals a template in this manner, a common growth direction normal to the extended chain nucleus and hence to the extrusion direction [47,60]. For this process to happen, the extended chains must still be present at the point of crystallisation and have not relaxed to a random coil configuration. The time between extension and crystallisation is critical here, and the nucleating effect of the GPN will shorten this time to a certain extent. Moreover, the templating effect of the GPN will have an equivalent effect: chain-folded lamellar crystals growing out from a surface rather than growing out in a radially symmetric manner from the row nucleus. Figure 4b shows an increase in the level of preferred orientation with GPN addition, however in GPN0.75 this level decreased. It might be thought that this is due to the poorer dispersion of the higher concentration of GPN. However, DSC measurement revealed a more-or-less linear increase in *T_c_* with GPN content. We attribute this fall off to the change of flow behaviour of the nanocomposites in the extruder die, which can be related to the changing rheology of the nanocomposites with GPN content. Correlation functions and structural parameters of the lamellar crystals presented in Figure 4a reveal that the addition of GPN serves to modify the preferred orientation of the lamellar crystals [61]. Moreover, the level of crystallinity drops for the nanocomposite with the highest fraction of GPN, where the filler may serve to inhibit the growth of crystals.

Mechanical properties of PCL and PCL/PGN scaffolds were analysed under compression, since this is one of the most important requirements for hard-tissue-replacement biostructures. All scaffolds displayed similar stress–strain curves with three different zones: an initial stiff mechanical response (linear elastic or Hookean region) followed by a plastic region after reaching the yield stress, ending with an increase in stiffness due to the compaction of the scaffold fibres [50,62]. These curves are similar to the results acquired in other works with highly porous polymeric scaffolds [63,64,65]. Results suggest that during the extrusion process of the scaffolds there occurs improvement in the alignment of graphene layers in the PCL filaments, which is corroborated by the SAXS patterns obtained. In other words, the improvement of the mechanical properties upon the addition of GPN can be attributed to the lamellar structure of the GPN, which enhances polymer–filler interactions between the filler and polymer matrix, thereby leading to better stress transfer [66,67]. On the other hand, the decrease of mechanical performance in the GPN0.75 structures could be related to the lower dispersion of GPN throughout the sample, as referenced in other works in the literature [12], but that would be contrary to the nucleating behaviour, which is more or less linear with GPN content. We identify that there is a direct correlation between lamellar crystal orientation and the mechanical performance of the scaffolds (Figure 4b,d), in which lamellar crystals perpendicularly oriented result in better compressive responses. Increasing the GPN to 0.75% results in the development of less orientation of the graphene during extrusion, and, although the graphene nucleates the PCL crystallisation, the lower graphene orientation leads to an overall lower preferred lamellar orientation.

In recent years, the research on GPN for biomedical applications has undergone exponential growth, mainly due to its electroconductivity, which is highly promising for the regeneration of electroactive tissues such as bone [68]. Regarding electrical conductivity measurements of the strands within the composite, clearly the fraction of GPN added or the dispersion was insufficient to yield a percolative network. Low percolation thresholds are observed with solution-mixed composites, as was performed in this work. He and Tjong [69] have prepared percolative composites with graphene oxide and PVDF with a volume fraction of graphene oxide of 0.31% *w*/*w*. One of the lowest values of percolation threshold observed for graphene composites prepared by melt mixing is the work of Gkourmpis et al. [70]. Increasing the graphene content to achieve percolation may lead to poor dispersion, and the methodology of Sayyar and colleagues [49], which involves covalently attaching the graphene to the PCL, is a very interesting approach and yields high conductivities with excellent dispersion. Wang and co-workers reported some in vivo cytotoxic responses with the addition of graphene oxide [71]. Another work reports that cell response to GPN depends on material surface, percentage, and synthesis methods, but also cell type used [72]. Accordingly, in vitro cytotoxicity assay following the ISO 10993-5 standards was performed to assure the biocompatibility of the produced scaffolds. Regarding L929 fibroblast proliferation tests, it was possible to observe that L929 fibroblasts presented low levels of cell adhesion to the scaffolds, which may be related to the hydrophobic nature of the PCL, which is in accordance with other results of our group [73], but with a high proliferation capacity.

Worldwide efforts have been made in order to achieve the best cell–material combination for a proper biological response in vivo to promote tissue regeneration. Despite all those efforts, there is not an effective answer yet. To achieve such an ideal combination, it is important to really understand the impact of cells on material performance and the material’s impact on cell behaviour (e.g., adhesion, proliferation, and/or differentiation). One of the key aspects relates to the ability of cells to proliferate and migrate throughout the whole scaffold structure to obtain uniform cell distribution. Due to their proliferation and migration capacity, MSCs present themselves as a good choice to evaluate the scaffold’s biological performance. For this, a three-week experiment was performed, culturing BM MSCs on the different scaffold experimental groups. BM MSCs presented the same behaviour as L929 fibroblasts regarding cell adhesion and proliferation. Moreover, the results obtained proved the great migration capacity of these cells. Although there were no significant statistical differences between PCL and PCL/GPN structures after three weeks (Figure 7), the results for 7 and 14 days showed an increased number of cells with the higher levels of GPN. The addition of GPN to PCL can result in an increase in its hydrophilic nature, but this also depends on the thickness of the graphene nanoflake. Hong and co-workers [74] have shown that the surface property of one or two layers of graphene can be influenced by the underlying environment. We can speculate that the addition of GPN to the PCL changes its surface properties to increase cell adhesion or some other properties. Of course, the graphene may influence the surface properties directly or by changing the morphology of the PCL matrix. An aligned system of lamellae could lead to a more hydrophilic surface by exposing the ester groups at the material surface. As the number of cells increases with time (and particularly because of the natural migration of BM MSCs), these surface effects become less significant for the increased cell population. The availability of the ester groups at the surface will also have a significant impact on the rate of hydrolysis necessary for degradation. We note that LIVE/DEAD staining revealed some level of cell death with the higher GPN content when compared to the other conditions. A possible explanation for this observation may be related to some GPN release from the scaffold in such a concentration that it impairs cell viability. However, in accordance with previous studies, the regular morphology and high proliferation/migration capacity demonstrated by the BM MSCs cultured on 3D PCL/GPN scaffolds (Figure 8) support their suitable application in tissue-engineering strategies, particularly focusing on bone regeneration [24,75,76].

## 5. Conclusions

In this study, we developed PCL scaffolds reinforced with GPN, which revealed significant enhancements in properties over the pristine polymer. Overall, throughout this work it was possible to infer the correlation between the addition of GPN and lamellar crystal orientation, which reveals a great impact on the scaffold’s mechanical properties and, finally, to study its impact on cell behaviour. According to the results obtained in this work, small amounts of GPN can serve as structural reinforcement to PCL scaffolds, improving their mechanical performance, in particular to a maximum of 0.5% of GPN content. Higher contents have a negative impact on scaffold mechanical performance under compression. This study also exposes the complexity of modifying the polymer matrix with nanoparticles, which will also lead to other effects. The potential of PCL/GPN as a biocompatible scaffold for TE strategies was further confirmed by high viabilities and proliferation capacity observed for both L929 mouse fibroblasts and human BM MSCs. In conclusion, the excellent processability and properties of this composite material make it highly promising for TE applications targeting the regeneration of hard, electroactive tissues, such as bone.

## Figures and Tables

**Table 1:** D scaffold design and AM extrusion-based processing parameters.

Design parameters	Filament gap	FG	[µm]	350
	Filament distance	FD	[µm]	650
	Filament width	FW	[µm]	300
	Slice thickness	ST	[µm]	280
Processing parameters	Melting temperature	MT	[°C]	80
	Deposition velocity	DV	[mm/min]	480
	Screw rotation velocity	SRV	[rpm]	30
	Nozzle diameter	ND	[µm]	300

**Table 2:** Thermophysical properties of PCL-based composites obtained from DSC and TGA: *T_c_*; *T_m_*; Δ*H_m_*; *X_c_*; Δ*M*; *P DTG*. Two-way ANOVA, uncorrected Fisher’s LSD (*n* = 3), multiple comparisons to a control (PCL). Statistical differences: ** *p* < 0.01; *** *p* < 0.001.

	Pre-Processing				Post-Processing			
	PCL	GPN0.25	GPN0.50	GPN0.75	PCL	GPN0.25	GPN0.50	GPN0.75
**T_c_** [°C]	35.84 ± 1.65	*** 39.67 ± 0.18	*** 39.68 ± 0.01	*** 39.84 ± 0.18	38.20 ± 0.11	*** 40.68 ± 0.01	*** 41.59 ± 0.12	*** 42.54 ± 0.06
**T_m_** [°C]	58.84 ± 0.27	59.06 ± 0.05	59.24 ± 0.06	59.26 ± 0.05	59.06 ± 0.04	59.29 ± 0.28	59.30 ± 0.01	59.45 ± 0.08
**ΔH_m_** [J/g]	58.26 ± 031	58.40 ± 1.18	** 59.60 ± 1.60	58.41 ± 0.33	58.08 ± 0.98	*** 61.00 ± 1.45	*** 62.96 ± 0.63	*** 62.95 ± 0.75
**X_c_**	0.42 ± 0.00	0.42 ± 0.01	0.43 ± 0.01	0.42 ± 0.00	0.42 ± 0.01	0.44 ± 0.01	0.45 ± 0.01	0.45 ± 0.01
**ΔM** [%]	99.14 ± 0.30	99.14 ± 0.85	99.39 ± 0.54	99.50 ± 0.32	99.58 ± 0.44	98.84 ± 0.59	99.43 ± 0.56	*** 98.06 ± 0.88
**P DTG** [°C]	410.22 ± 0.15	*** 406.73 ± 0.25	410.23 ± 0.12	409.79 ± 0.42	411.14 ± 0.52	411.24 ± 0.49	411.19 ± 0.58	411.43 ± 0.51

## Data Availability

The data that support the findings of this study are available from the corresponding author on request.

## Supplementary Materials

The following supporting information can be downloaded at: https://www.mdpi.com/article/10.3390/polym14091669/s1, Figure S1: Bone marrow MSC proliferation assay—SEM analysis (Day 21), magnification: 150×.
