# Supplementary material for: Additive Manufactured Poly(ε-caprolactone)-graphene Scaffolds: Lamellar Crystal Orientation, Mechanical Properties and Biological Performance
Source: Polymers (Basel). 2022 Apr 20;14(9):1669. doi: 10.3390/polym14091669 (PMC9101196; doi:10.3390/polym14091669)
Supplement: Supplementary file 1 [file polymers-14-01669-s001.zip › polymers-1625613-supplementary.pdf]

Supplementary material to:

Additive manufactured poly(ε-caprolactone)-graphene scaffolds: lamellar crystal orientation, mechanical properties and biological performance

Sara Biscaia[1], João C. Silva[1,2,3], Carla Moura[1], Tânia Viana[1], Ana Tojeira[1], Geoffrey R. Mitchell[1], Paula Pascoal-Faria[1], Frederico Castelo Ferreira[2,3] and Nuno Alves[1*]

[1] Centre for Rapid and Sustainable Product Development, Polytechnic Institute of Leiria, Portugal; sara.biscaia@ipleiria.pt (S.B.); carla.moura@ipleiria.pt (C.M.); taniaviana@gmail.com (T.V.); anatojeira88@gmail.com (A.T.); geoffrey.mitchell@ipleiria.pt (G.R.M.), paula.faria@ipleiria.pt (P.P.F.); nuno.alves@ipleiria.pt (N.A.)
[2] Department of Bioengineering and iBB - Institute for Bioengineering and Biosciences, Instituto Superior Técnico, Universidade de Lisboa, Av. Rovisco Pais, Lisboa 1049-001, Portugal; joao.f.da.silva@tecnico.ulisboa.pt (J.C.S.); frederico.ferreira@tecnico.ulisboa.pt (F.C.F.)
3 Associate Laboratory i4HB-Institute for Health and Bioeconomy, Instituto Superior Técnico, Universidade de Lisboa, Av. Rovisco Pais, Lisboa 1049-001, Portugal

*Correspondence: nuno.alves@ipleiria.pt

**Cell Culture** (Day 21)	**PCL**	**GPN0.25**	**GPN0.50**	**GPN0.75**
**SEM**	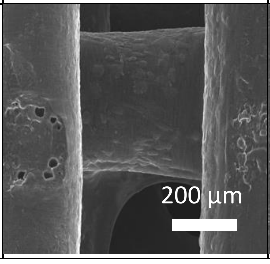	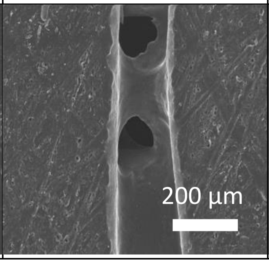	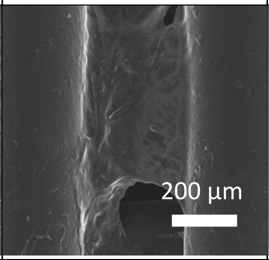	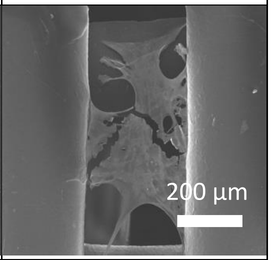

Figure S1 Bone-marrow MSCs proliferation assay – SEM analysis (Day 21), magnification:150x.
